# Reinforced Polymer Composites

**DOI:** 10.3390/polym13040564

**Published:** 2021-02-13

**Authors:** Victor V. Tcherdyntsev

**Affiliations:** Laboratory of Functional Polymer Materials, National University of Science and Technology “MISIS”, Leninskii prosp, 4, 119049 Moscow, Russia; vvch@misis.ru; Tel.: +7-9104002369

The development of modern technology requires the elaboration of new materials with improved operational and technological properties. At the same time, many of the currently known natural and artificial materials no longer meet the increasing requirements. The discovery of fundamentally new materials is an extremely rare phenomenon, which indicates that the overwhelming majority of “simple” materials have already been discovered, and there is no need to expect great achievements in this direction. Therefore, the main direction in the development of new materials is now the creation and improvement of composite materials.

The main advantages of composites over traditional types of materials are:

- a unique combination of properties (strength, deformation, elastic, electrical, frictional, thermophysical, and others), which cannot be achieved for “simple” materials;
- the ability to control the composites’ properties over a wide range by simply changing their composition and production conditions.

In polymer composites reinforced with dispersive fillers, the matrix is the main element undergoing the mechanical loading, and the main purpose of introducing dispersed particles usually is to increase the elastic modulus of the material. Dispersed filling is also used to improve the thermophysical characteristics, electrical and magnetic properties, to reduce friction wear, and to reduce the flammability of the material. In the case of composites reinforced with continuous fillers, both the matrix and reinforcers play an important role in the composite mechanical behavior, and the characteristics of composites are associated strongly with the ability of the interface to transfer the mechanical loading from the matrix to reinforcers. Initially, the main goal of polymer filling was to reduce the cost of the material by using an inexpensive filler. Today, reinforced polymer composites are some of the most numerous and diverse types of materials that are promising for use in various fields of science and technology, where high requirements are imposed on the physical, mechanical, and tribological characteristics.

In industry, some polymers have become widespread as antifriction wear-resistant materials that can work in dry friction conditions and in aggressive environments. Antifriction polymers act as substitutes for such traditional materials as bronze, brass, steel, and antifriction cast iron. The combination of antifriction properties with high biocompatibility allows the use of polymers in the creation of implants in the musculoskeletal system. However, polymers materials in their original form have a number of disadvantages, which include low strength and hardness and low operating temperature, which significantly limits their use. To improve these properties, researchers are attempting to reinforce polymers with various fillers and create composite materials based on them. A special class among composite materials is nanocomposites, in which, due to the use of nanosized particles, a more uniform distribution of the reinforcing element in the matrix and strong interfacial interactions between the polymer and the filler are achieved. Nanoparticles are capable of influencing the crystallization mechanism of polymers, acting as a nucleating agent, on the surface of which the crystalline phase nucleates. Depending on the size, shape, and nucleation density of nanoparticles, the formation of various supramolecular structures in polymers is possible.

To obtain high-strength composites, it is necessary to provide a high transfer capacity of the load from the matrix to the filler. This ability is determined by the level of adhesion of the filler to the polymer matrix. The adhesion of the filler to the polymer can be achieved by:

- chemical interaction;
- mechanical adhesion of the filler and matrix;
- electrostatic and van der Waals forces.

The adhesion due to the mechanical adhesion of the filler and the matrix depends on the geometry of the filler surface and the properties of the polymer, which include the chemical structure of the polymer, the regularity of the molecular structure, conformational characteristics, and branching of polymer chains. Furthermore, mechanical adhesion is affected by the difference in the thermophysical properties of materials. For example, during the cooling of a composite material, the difference between the coefficients of the linear thermal expansion of the polymer and the filler can lead to the formation of residual thermal expansions. In the absence of chemical and mechanical interactions between the filler and the polymer matrix, the energy for pulling the filler out of the polymer is composed of electrostatic and van der Waals forces, which provide adhesive bonding.

The hardening mechanisms of reinforcing fillers are different and primarily depend on the type of filler. For example, when reinforced with continuous fibers, an increase in strength is achieved due to the redistribution of the load from the matrix to the fiber. The fiber takes on all the mechanical load, and accordingly, the strength of the composite is determined by the strength of the reinforcing fiber. The role of the matrix is to redistribute stresses between fibers and form a single whole composite.

This Special Issue consisting of 21 articles, including three review papers, written by research groups of experts in the field, considers recent research on reinforced polymer composites. First of all, it should be noted that all three review papers relate to the fiber reinforced polymer composites, which are a real hot topic in the field. Rajak et al. [1] give a general review of the present state of the fiber reinforced polymer composites. Classifications of composites along with the properties of their constituent elements are analyzed. Depending on the reinforcing fiber nature, such composites may be divided into synthetic and natural fiber reinforced ones. Synthetic fibers, such as carbon, glass, or basalt, provide more stiffness, while natural fibers, such as jute, flax, bamboo, kenaf, and other, are inexpensive and biodegradable, making them environmentally friendly. Due to the modern requirements of the environmental safety of the processes of both materials’ manufacturing and the effective recycling or utilization of materials at the end of the life cycle, using natural materials as reinforcers for polymer composites is a second hot topic in the field.

As is noted in [1], to acquire the benefits of design flexibility and recycling possibilities, natural reinforcers can be hybridized with small amounts of synthetic fibers to make them more desirable for technical applications. One case of such hybrid composites, namely kenaf/glass reinforced ones, is considered in the review paper of Nadzri et al. [2]. It is summarized that reinforcing of kenaf fiber containing composites, which have great potential in automotive application, due to being lightweight, eco-friendly, and low-cost, with glass fiber, is a good way to enhance such composites because glass fiber has better mechanical and impact properties than kenaf fiber. It is noted that the optimum fiber content and fiber orientation in such composites is between 30% and 40% and 90°, respectively. Fiber surface modification also helps to improve the properties of kenaf/glass hybrid composites.

Mohamed et al. [3] review recent advances in the development of glass and carbon fiber reinforced polymer composite laminates used in structurally deficient flat slab floor systems to enhance the two-way shear capacity, flexural strength, stiffness, and ductility. It is summarized that vertically placed fiber reinforced polymer sheets/laminae/strips are more effective in resisting two-way shear force. It is noted that further research is needed to develop models and clear design guidelines for vertically placed fiber reinforced polymer retrofitting strips that considers the pattern, spacing, material, and concrete properties. Additionally, the investigation of larger size specimens to predict the response of fiber reinforced polymer retrofitted slabs is required.

Looking through 18 regular research papers, published in this Special Issue, one can see that most of them relate to the above-mentioned hot topics. Among them, nine papers relate to polymer composites reinforced with synthetic fibers. Slonov and co-workers [4] study the effect of oligophenylene sulfone and polycarbonate additions on the rheological, mechanical, and thermal properties of polyetherimide reinforced with short carbon fibers. It is revealed that the addition of oligophenylene sulfone and polycarbonate significantly reduces the melt viscosity of polyetherimide based carbon filled composites, acting as a temporary plasticizer. The addition of oligophenylene sulfone results in a significant increase in the elastic modulus and strength of composites, whereas the introduction of polycarbonate leads to a decrease in toughness, while the elastic modulus and strength remain at the level of the initial composite. The introduction of both oligophenylene sulfone and polycarbonate leads to an increase in carbon fiber length in comparison with a composite without modifiers. For composites modified with oligophenylene sulfone, a higher adhesive interaction of the polymer matrix with the surface of the carbon fiber is observed, and the thermal stability and heat resistance of composites with oligophenylene sulfone melts are found to be significantly higher than for composites modified with polycarbonate.

Tian et al. [5] also use short carbon fiber as reinforcers for polymer composite; however, in this case, the modification is performed by the coating of the carbon fiber surface with a polydopamine layer. They prepare a high-performance rubber composite by mixing of dopamine-modified short carbon fiber with natural latex. It is observed that uniform and widely covered polydopamine coatings are formed on the carbon as a result of the modification, which significantly improves the interface adhesion between the carbon fiber and the rubber matrix. The modification of carbon fiber in the solution with the concentration of dopamine of 1.5 g/L for 6 h shows the best results among the treatment routes used. Natural fiber latex based composites reinforced with such carbon fibers show excellent thermal conductivity and dynamic mechanical properties, and their tensile strength is 10.6% higher than those of the composites containing unmodified fibers.

Zhu and coauthors [6] compare the wear resistance behavior of bearing bushes made of polyetheretherketone (PEEK), 30 wt % carbon fiber reinforced PEEK, 30 wt % glass fiber reinforced PEEK, and each 10 wt % of polytetrafluoroethylene, graphite, and carbon fiber filled PEEK. It is found that due to low thermal conductivity, unfilled PEEK and glass fiber reinforced PEEK present the much lowest articulating cycles to failure. The presence of graphite and polytetrafluoroethylene in the PEEK matrix not only reduces the shear force at the interface, but also minimizes the temperature increase in the bulk material. The wear mass loss of each 10 wt % of polytetrafluoroethylene, graphite, and carbon fiber filled PEEK is found to be 0.13 × 10^−6^ mm^3^/Nm compared with 4.33 × 10^−6^ mm^3^/Nm for PEEK filled with 20 wt % of carbon fiber. It is concluded that bushes made of PEEK composite formulated with polytetrafluoroethylene, graphite, and carbon fiber exhibit low friction, self-lubrication, and low temperature rise and therefore present superior bearing properties, including enhanced bearing life and reduced energy consumption in machinery.

In [7], the formation of polysulfone based composites reinforced with carbon fiber fabric via the solution impregnation method using N-methyl-2-pyrrolidone as the solvent is reported. To improve the adhesion between the polymer matrix and carbon fibers, thermal oxidation of carbon fibers is carried out. Such oxidation allows for a change in the carbon fibers’ surface chemical composition, and a greater number of functional groups on the surface appear as a result of thermal oxidation, resulting in a strong bond between the polysulfone matrix and carbon fiber. The in-plane shear strength value of polysulfone reinforced with modified carbon fibers increases by more than 1.5 times compared with the composites containing untreated carbon fibers. Surface modification of carbon fibers results also in noticeable improvement in the flexural and tensile properties, as well as in the thermal stability of polysulfone based composites. Sherif et al. use the same solution impregnation technique to obtain polyethersulfone [8] and polysulfone [9] based composites reinforced with glass fiber fabrics. The preheating of glass fibers is used to remove the sizing coating of fabrics and to improve the adhesion between the matrix and reinforcer. It is shown that glass fiber preheating allows increasing the mechanical properties of composites by 20–40%. For both polymer matrices used, the best mechanical properties are achieved at a glass fiber-to-polymer weight ratio of 70/30.

Xu and co-workers [10] made a composite of the commercial proton-exchange Nafion membrane specially fabricated via electrospinning silica nanofibers with a three-dimensional network structure. The proton conductivity of the silica nanofiber–Nafion composite membrane at 110 °C is found to be almost doubled compared with that of a pristine Nafion membrane, while the mechanical stability of the composite Nafion membrane is enhanced by 44%. It is found that the silica nanofiber-Nafion composite membrane exhibits great high-temperature fuel cell performance with a 118 mW/cm^2^ power density at low humidity, which is 38% higher than that of the pristine Nafion membrane.

Mao and co-workers [11] report investigations of the effect of the surface modification of polyethylene terephthalate fibers on their adhesion to a polypropylene matrix. Polyethylene terephthalate fiber is modified through solution dip-coating using a novel synthesized tetraethyl orthosilicate/silane coupling agent KH550/polypropylene maleic anhydride graft hybrid. As a result of such treatment, SiO_2_ particles are designed to link with the maleated polypropylene molecular chain through silane coupling agent KH550, which forms an organic-inorganic film by the grain structure on the surface of polyethylene terephthalate fiber. As a result, the bending strength and modulus of the polypropylene reinforced with modified polyethylene terephthalate fiber increase by 21 and 34%, respectively, compared to the untreated fiber-filled composite.

Huang et al. [12] apply both computer-aided engineering simulation and experimental methods to investigate the glass fiber feature in a co-injection molding system. Fiber orientation distributions and their influence on the tensile properties for the single-shot and co-injection molding are discovered. Results show that based on the 60:40 skin/core ratio and the same materials, the tensile properties of the co-injection system, including the tensile stress and modulus, are a little weaker than those of the single-shot system. To discover and verify the influence of the fiber orientation features, the fiber orientation distributions of both the co-injection and single-shot systems are observed using micro-computerized tomography technology to scan the internal structures. It is found that the fiber orientation tensor in the flow direction of the co-injection system is about 89% of that of the single-shot system in the testing condition because the co-injection part has lower tensile properties.

Two papers in this Special Issue cover polymer composites reinforced with natural fibers. Yeh and Yang [13] test four types of waste bamboo fibers, differing in plant species, as fillers in polypropylene based composites. It is found that the composites reinforced with thorny bamboo fibers (*Bambusa stenostachya*) exhibit the highest moisture content and water absorption rate due the high hemicellulose content in these fibers, while the composites reinforced with Makino bamboo fibers (*Phyllostachys makinoi*) possess better tensile properties due to the high crystallinity and high lignin content of these fibers.

Roy et al. [14] investigate the effect of jute fiber surface treatment by alkali, stearic acid, or silane on the properties of natural rubber based composites. They show that combined alkali/silane treatment is the most efficient surface treatment method to develop strong interfacial adhesion between the natural rubber matrix and jute fibers. Composites reinforced with jute fibers modified by combined alkali/silane treatment show considerably higher torque difference, tensile modulus, hardness, and tensile strength as compared to either untreated or other surface treated jute fiber filled natural rubber.

The topic of polymer composites reinforced with hybrid fillers containing both synthetic and natural components in this Special Issue is covered by four papers. Shah and co-workers [15] investigate the impact behavior of epoxy based composites reinforced with woven glass fabric and bamboo powder and compare the obtained results with the data on the neat epoxy and epoxy reinforced with short bamboo fibers. Woven glass fibers are embedded at the outer most top and bottom layer of the bamboo powder filled epoxy composites, producing sandwich structured hybrid composites through lay-up and molding techniques. A significant improvement is observed with the inclusion of woven glass fibers in the composites. The non-hybrid composites break into pieces during the highest impact energy that is applied, while the hybrid composites experience only perforation, and the structure does not totally break. The non-hybrid composites can withstand an impact energy up to 10 J, while the hybrid composites show total failure at 35 J.

Calabrese et al. [16], using the vacuum-assisted resin infusion technique, prepare epoxy based composites reinforced with six layers of 2 × 2 twill weave woven flax fabric. Three layers of plain weave woven glass fabrics are used as external fiber reinforced skins, and the number of fully reinforced layers is 12. Samples with varying geometrical joint configurations are exposed to a salt-fog spray test up to 60 aging days, and the effect of the salt-fog environment on the mechanical behavior of the pinned hybrid glass-flax/epoxy composites is evaluated. A noticeable modification of the damage mode with increase of the aging exposition time is evidenced, with a reduction of the bearing phenomenon, thus favoring premature and catastrophic mechanisms such as shear out and net tension. As a consequence, the effective mechanical durability of the mechanical joint is limited.

Ibrahim et al. [17] investigate the isolation of lignin from empty oil palm fruit bunch fibers using formic acid at different concentrations. The isolated organosolv lignin is modified with graphene nanoplatelets and used as the filler reinforcement for photo-curable polyurethane in stereolithography 3D printing. Reinforcing of polyurethane with 0.6 wt % of graphene modified lignin provides tremendous enhancement of the hardness at 92.49 MPa, which means a 238% increment from the unmodified photo-curable polyurethane resin. Moreover, this composite shows higher tensile strength and resistance against the deformation of the material.

Hayeemasae and co-workers [18] utilize modified palm stearin as a mixed compatibilizer with maleated natural rubber for natural rubbed based composites reinforced with halloysite nanotubes. The main idea of the study is to use chip natural product, such as palm stearin, instead of silanes, which are considered to be expensive and require a high mixing temperature to obtain effective silanization. It is found that the overall properties of composites based on natural rubber and halloysite nanotubes are clearly improved by adding maleated natural rubber/modified palm stearin as a dual compatibilizer. Both maleated natural rubber and modified palm stearin have special functional groups that form hydrogen bonds with the hydroxyl groups on halloysite surfaces. Moreover, modified palm stearin can also promote the dispersion of halloysite nanotubes filler in the matrix due to its waxy character. It is observed that improvement in the filler-matrix interactions due to the above-mentioned dual compatibilizer addition results in the increase in the composites’ mechanical properties, such as the tensile strength, modulus, and tear strength.

The remaining three papers in this Special Issue do not relate to polymer composites reinforced with fiber fillers. Chang et al. [19] investigate the effect of combining two approaches, namely the addition of nanoparticles and the crosslinking of two different polymers to create double-network hydrogels, on the mechanical properties of hydrogels. It is evaluated that the introduction of nanomaterials into the hydrogel network may allow the consideration of double-network hydrogels for new applications such as self-healing, shape memory, 3D printing, and dye removal. A mechanistic insight into the role of nanoparticles in the mechanical properties of double-network hydrogels is provided, including the elastic moduli and swellability. The existence of a “global” saturation point for double-network hydrogel nanocomposites is observed, beyond which it becomes less plausible to enhance the elastic modulus by simply increasing the concentration of the second network’s hydrogel or nanoparticles.

The effect of Na_2_CO_3_ addition on the structure and properties of polyvinyl alcohol/carboxyl methyl cellulose sodium composite films is investigated in [20]. It is observed that the presence of Na_2_CO_3_ results in the hydrolysis of the vinyl acetate group of polyvinyl alcohol and in the decrease in the crystallinity of the composite film. At the same time, as the Na_2_CO_3_ content increases, more ester groups are hydrolyzed, and the amount of hydroxyl groups increases, which generates more intermolecular hydrogen bonds and, thus, increases the melting temperature of the composite. Additionally, the increase in the Na_2_CO_3_ content results in a significant increase in the water sorption properties of the composite films. The elaborated polyvinyl alcohol/carboxyl methyl cellulose sodium/Na_2_CO_3_ composites may have prospective applications as biodegradable superabsorbent resins and polymer electrolytes.

Finally, Lei et al. [21] study polymer blends containing nitrile rubber, brominated butyl rubber, and ethylene-vinyl acetate copolymer. It is shown that such blends exhibit excellent vulcanization plateaus and mechanical properties. Ethylene-vinyl acetate copolymer is observed to be the optimal polymer for improving the compatibility of the nitrile rubber/brominated butyl rubber blend. Hot air thermal aging tests show that the blends have good stability. Besides the studied blends, nitrile rubber/brominated butyl rubber/ethylene-vinyl acetate copolymer with a 50/50/30 blend ratio is found to be a comparatively ideal material for the purpose of high-damping isolation bearings. As the formation of the polymer blend can be applied to optimize the properties of polymer composites matrices, the obtained results may be of interest for the elaboration of advanced reinforced polymer composites.

## Data Availability

No new data were created or analyzed in this study. Data sharing is not applicable to this article.
